# Mechanism exploration of di(2-ethylhexyl) phthalate (DEHP)-induced hepatocellular carcinoma via network toxicology and molecular docking analysis

**DOI:** 10.1007/s12672-026-05081-2

**Published:** 2026-04-24

**Authors:** Tao Wang, Yi Zhou, Bin Ge, Dacai Gong, Peng Chen

**Affiliations:** 1 Department of Clinical Laboratory, The Third People’s Hospital of Wenjiang District, Chengdu, Sichuan China; 2 Department of Clinical Laboratory, Pidu District People’s Hospital, The 3RD Affiliated Hospital of Chengdu Medical College, Chengdu, Sichuan China

**Keywords:** Di(2-ethylhexyl) phthalate, Hepatocellular carcinoma, Network toxicology, Metabolic reprogramming, Immune microenvironment, Machine learning

## Abstract

**Background:**

Hepatocellular carcinoma (HCC) is one of the leading causes of cancer-related mortality worldwide, and its development is closely associated with metabolic dysregulation. Di(2-ethylhexyl) phthalate (DEHP), a widely used plasticizer, has been implicated in hepatotoxicity and carcinogenesis; however, the molecular mechanisms linking DEHP exposure to HCC progression, particularly through metabolic reprogramming and immune microenvironment alterations, remain poorly understood.

**Methods:**

In this study, we integrated two GEO HCC transcriptomic datasets and applied differential expression analysis and weighted gene co-expression network analysis (WGCNA) to identify DEHP-related gene modules. Potential DEHP targets were predicted using ChEMBL, PharmMapper, and SwissTargetPrediction. Functional enrichment and protein–protein interaction network analyses were performed, followed by hub gene identification using multiple machine learning algorithms, including LASSO, SVM-RFE, and Random Forest. Immune cell infiltration was evaluated using CIBERSORT, and molecular docking combined was conducted to validate DEHP–protein interactions.

**Results:**

Integration of DEHP target predictions and HCC transcriptomic analyses identified 39 overlapping genes potentially associated with DEHP-related HCC. Functional enrichment analysis revealed significant involvement in xenobiotic metabolism, fatty acid degradation, PPAR signaling, and peroxisomal pathways. Network topology and machine learning approaches consistently highlighted ACACB, ADH4, and PCK1 as core hub genes, all of which showed significant differential expression between tumor and normal tissues and demonstrated strong diagnostic performance (AUC > 0.87). Immune deconvolution analysis revealed DEHP-associated immune landscape alterations, with hub gene expression exhibiting significant correlations with multiple immune cell subsets. Molecular docking indicated favorable binding between DEHP and the three hub proteins.

**Conclusions:**

This study provides systematic evidence that DEHP may contribute to HCC development through dysregulation of metabolic enzymes and remodeling of the immune microenvironment. ACACB, ADH4, and PCK1 may serve as potential biomarkers and mechanistic links between environmental DEHP exposure and hepatocarcinogenesis.

**Supplementary Information:**

The online version contains supplementary material available at 10.1007/s12672-026-05081-2.

## Introduction

Hepatocellular carcinoma (HCC) remains a leading cause of cancer-related mortality globally, with its complex etiology involving viral infections, metabolic disorders, and environmental exposures. Emerging evidence increasingly implicates metabolic reprogramming as a fundamental characteristic of HCC pathogenesis, whereby cancer cells undergo coordinated alterations in energy metabolism, biosynthetic pathways, and redox homeostasis to support uncontrolled proliferation and survival in nutrient-deprived microenvironments [1, 2]. Among the metabolic alterations characteristic of HCC, dysregulated lipid metabolism has garnered particular attention. Aberrant fatty acid synthesis and oxidation, mediated by enzymes such as acetyl-CoA carboxylases (ACCs), fatty acid synthase (FASN), and carnitine palmitoyltransferases (CPTs), contribute to lipid accumulation, membrane remodeling, and energy provision in hepatocellular carcinoma cells [3, 4].Concurrently, alterations in gluconeogenesis and glycolysis—governed by key regulatory enzymes including phosphoenolpyruvate carboxykinase (PCK1), pyruvate kinase (PKM2), and hexokinases—facilitate metabolic flexibility and biosynthetic precursor generation [5] Additionally, xenobiotic metabolism pathways involving cytochrome P450 enzymes and alcohol dehydrogenases serve dual roles in detoxification and metabolic regulation, forming critical nodes that link environmental exposures to endogenous metabolic perturbations [6].

Di(2-ethylhexyl) phthalate (DEHP) represents one of the most widely used plasticizers globally, with ubiquitous human exposure occurring through food packaging, medical devices, and consumer products [7]. Mounting epidemiological and experimental evidence links DEHP exposure to hepatotoxicity, metabolic disorders, and carcinogenesis, with the liver serving as the primary organ for DEHP metabolism and detoxification [8]. Upon entering the body, DEHP undergoes rapid hydrolysis to mono(2-ethylhexyl) phthalate (MEHP), which subsequently activates peroxisome proliferator-activated receptors and disrupts multiple metabolic pathways. The mechanistic basis for DEHP-induced hepatocarcinogenesis involves oxidative stress generation, epigenetic modifications, and disruption of lipid homeostasis, ultimately promoting cellular transformation and tumor development [9, 10]. Recent studies have revealed that DEHP exposure can alter the expression of key metabolic enzymes, including those involved in fatty acid metabolism, steroid hormone biosynthesis, and xenobiotic processing [11]. However, the precise molecular mechanisms through which DEHP influences HCC development, particularly regarding metabolic enzyme dysregulation and immune microenvironment remodeling, remain incompletely understood.

Despite extensive independent research on hepatocellular carcinoma (HCC) pathogenesis and di-(2-ethylhexyl) phthalate (DEHP) toxicity, the molecular mechanisms linking DEHP exposure to HCC development—particularly through metabolic enzyme dysregulation and immune microenvironment remodeling—remain poorly understood. To address this knowledge gap, we employed an integrative bioinformatics and systems biology framework to generate testable hypotheses regarding potential DEHP-responsive genes and pathways in HCC. Through systematic integration of computational target prediction, transcriptomic profiling, co-expression network construction, machine learning-based feature selection, immune infiltration analysis, and molecular docking simulations, this study aims to identify candidate molecular links between environmental DEHP exposure and hepatocarcinogenesis. We hypothesize that DEHP exposure may contribute to HCC development through dysregulation of key metabolic enzymes involved in lipid metabolism, xenobiotic detoxification, and glucose homeostasis, which in turn may reshape the tumor immune microenvironment. If validated experimentally, the identified hub genes and pathways could serve as potential biomarkers for early detection of DEHP-associated hepatic metabolic dysfunction and may represent novel therapeutic intervention points. By prioritizing candidate genes through multiple complementary computational approaches, this work provides a data-driven framework to guide future experimental investigations aimed at elucidating the molecular mechanisms underlying DEHP-induced hepatotoxicity and carcinogenesis.

## Materials and methods

### Acquisition of DEHP targets

The molecular structure of DEHP and Simplified Molecular Input Line Entry System (SMILES) “CCCCC(CC)COC(= O)C1 = CC = CC=C1C(= O)OCC(CC)CCCC” were obtained from the PubChem database [12]. Potential human protein targets were predicted with three complementary approaches to maximize recall and reduce method-specific bias: (1) ChEMBL (ligand–target bioactivity inference) [13], (2) PharmMapper [14](reverse pharmacophore mapping), and (3) SwissTargetPrediction (2D/3D similarity and statistical learning) [15]. Predicted targets were merged by UniProt gene symbols, deduplicated, and restricted to Homo sapiens entries. Database accession links and repository details are provided in the Data Availability section.

### Dataset acquisition and preprocessing

Two hepatocellular carcinoma (HCC) transcriptomic datasets (GSE36376 and GSE76427) were retrieved from the NCBI Gene Expression Omnibus (GEO) database, comprising a total of 245 control samples and 355 HCC samples. To minimize batch effects across datasets, a two-stage normalization pipeline was applied. First, quantile normalization was performed separately within each dataset to ensure comparable expression signal distributions among samples. Second, inter-dataset batch effects were corrected using the ComBat algorithm implemented in the sva package, which employs a parametric empirical Bayes framework to adjust batch-specific variations while preserving biological differences between HCC and control groups [16]. Principal component analysis (PCA) was conducted before and after batch correction to assess the effectiveness of technical artifact removal and the improvement in sample clustering according to biological condition. The batch-corrected and harmonized expression matrix was used for all subsequent downstream analyses. The accession numbers and full repository details for these datasets are provided in the Data Availability section.

### Identification of differentially expressed genes and WGCNA construction

Differentially expressed genes (DEGs) between hepatocellular carcinoma (HCC) tumor tissues and adjacent normal liver tissues were identified using the limma package, with a false discovery rate (FDR)–adjusted P value < 0.05 and |log2 fold change (FC)| > 1 (2-fold change) as the screening criteria, and the results were visualized using ggplot2. Subsequently, weighted gene co-expression network analysis (WGCNA) was performed based on the expression profiles of the identified DEGs to construct a scale-free gene co-expression network [17]. Hierarchical clustering was first applied to remove outlier samples, followed by selection of an appropriate soft-thresholding power to achieve scale-free topology (R² > 0.85). Gene modules were identified using topological overlap matrix (TOM)-based hierarchical clustering with dynamic tree cutting (minModuleSize = 30) and merged at a cut height of 0.25. Module–trait relationships were evaluated by correlation analysis, and significant modules were defined as those with |R| > 0.5 and *P* < 0.05. Hub genes within key modules were further identified based on high intramodular connectivity (kME > 0.8).

### Functional enrichment analysis

The interGenes were subjected to Gene Ontology (GO) and Kyoto Encyclopedia of Genes and Genomes (KEGG) pathway enrichment analyses using the clusterProfiler R package, with statistical significance defined as *P* < 0.05. The results were visualized with the ggplot2, circlize, and ComplexHeatmap R packages.

### Construction of PPI network and identification of HubGenes

Protein–protein interaction (PPI) relationships among the interGenes were analyzed using the Search Tool for the Retrieval of Interacting Genes/Proteins (STRING) database (https://string-db.org/). Isolated nodes were manually removed to retain biologically meaningful interactions. The resulting PPI network was subsequently visualized using Cytoscape version 3.10.3 (https://cytoscape.org/).

### Machine learning

To rigorously identify the most informative hub genes, three complementary machine learning strategies were applied. First, Least Absolute Shrinkage and Selection Operator (LASSO) regression was conducted using the glmnet package with a binomial model and 10-fold cross-validation to select the optimal λ value that minimized prediction error while eliminating redundant variables [18]. Second, Support Vector Machine–Recursive Feature Elimination (SVM-RFE) was implemented via the e1071 package with 10-fold cross-validation, iteratively discarding features with the lowest weights to identify the most discriminative genes while minimizing the root-mean-square error. Third, Random Forest analysis was performed using 500 decision trees to evaluate variable importance in the high-dimensional dataset, and genes with importance scores greater than 10 were retained as biologically meaningful features [19]. Genes consistently identified by all three methods—each leveraging distinct mathematical principles for feature selection—were defined as the final set of HCC–related genes. The overlap among the selected genes was illustrated using Venn diagrams to demonstrate consensus. This integrative, multi-algorithm approach enhanced both the statistical reliability and biological relevance of the gene selection process.

### Differential expression analysis and visualization

The expression levels of hub genes between control and case groups were compared using Student’s t-test. Gene expression distributions were visualized using boxplots with overlaid jittered points to display individual samples. Statistical significance was annotated directly on the plots. All visualizations were generated using the ggplot2 and ggpubr R packages.

### ROC analysis

Receiver operating characteristic (ROC) curve analysis was conducted to assess the diagnostic performance of hub genes. ROC curves and area under the curve (AUC) values were generated using the pROC package, with classification direction determined automatically and AUC values < 0.5 adjusted accordingly. The 95% confidence intervals of AUCs were estimated by bootstrap resampling, and both individual and combined ROC curves were visualized for comparative evaluation.

### Immune infiltration analysis

Immune cell infiltration in the microenvironment was evaluated using CIBERSORT (Cell-type Identification By Estimating Relative Subsets of RNA Transcripts), which defines 22 human immune cell types based on 547 signature genes and applies a linear support vector regression model for immune cell deconvolution [20]. The relative infiltration patterns of immune cells were visualized using bar plots and heatmaps. Spearman correlation analysis was performed to assess the relationships between hub genes and immune cell infiltration, as well as immune-related factors.

### 10 Molecular docking

Three-dimensional structures of the core target proteins were obtained from the Protein Data Bank, with preference given to high-resolution human crystal structures; high-confidence AlphaFold models were used when experimental structures were not available. Protein structures were preprocessed by removing crystallographic water molecules and co-factors, adding polar hydrogens, and assigning protonation states corresponding to physiological pH (7.4). The DEHP ligand was protonated and subjected to energy minimization prior to docking. Molecular docking was performed using AutoDock Vina, with docking grids positioned around predicted or experimentally validated binding sites [21]. Binding conformations with docking scores ≤ − 5.0 kcal/mol were considered to represent favorable interactions and were further examined for structural and chemical rationality using PyMOL. Selected protein–PET complexes were subsequently simulated by 100 ns all-atom molecular dynamics simulations in GROMACS 2022 employing the CHARMM36 force field and TIP3P explicit solvent model under constant temperature and pressure conditions.

## Results

### Identification of potential protein targets of DEHP

**Fig. 1:**
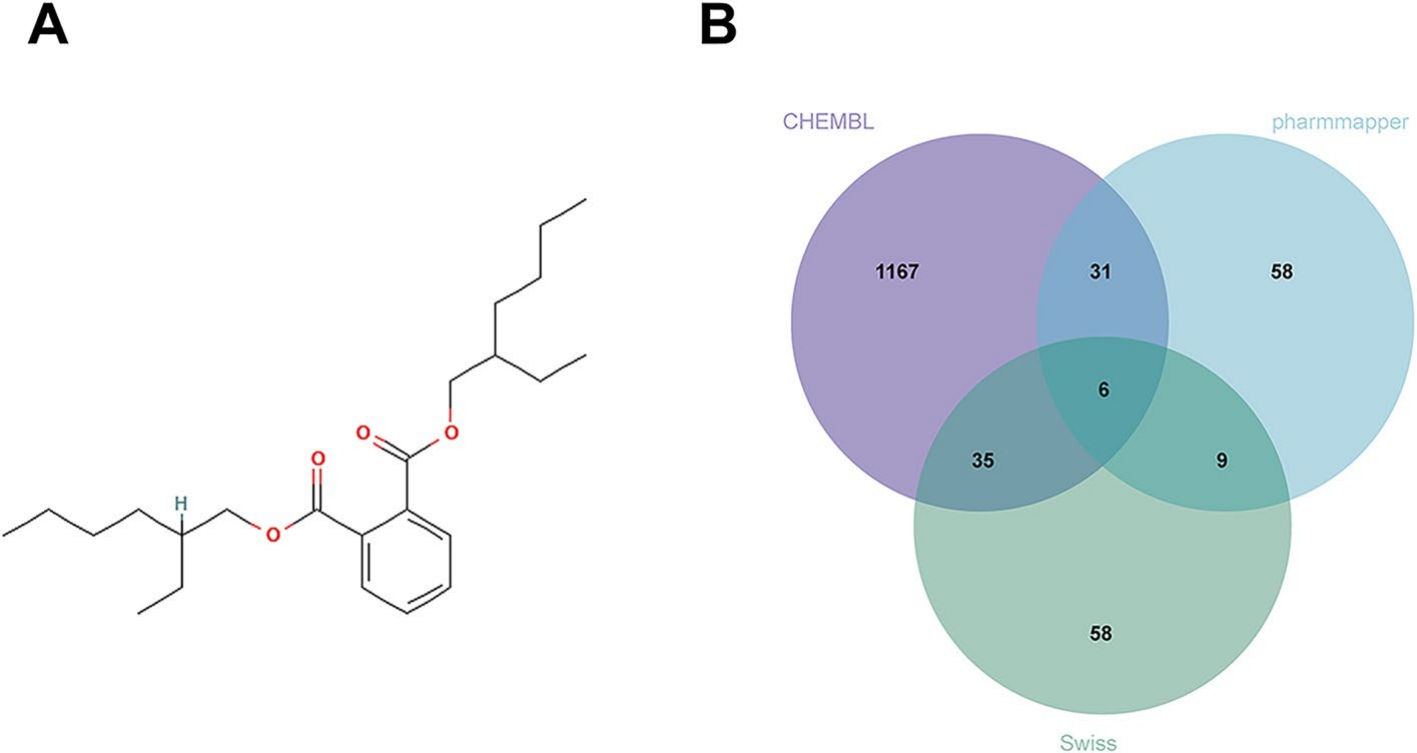
Structure of DEHP and Prediction of Potential Targets. **A** Chemical structure of DEHP obtained from the PubChem database. **B** Merged and de-duplicated target sets from ChEMBL, PharmMapper, and SwissTargetPrediction, identifying 1452 unique candidate targets

The molecular structure of di(2-ethylhexyl) phthalate (DEHP) was first obtained from the PubChem database (Fig. 1A). Potential molecular targets associated with DEHP exposure were then predicted using three complementary computational platforms: ChEMBL, PharmMapper, and SwissTargetPrediction. After integrating the outputs from these databases and eliminating redundant entries, a total of 1,452 unique candidate target proteins were identified (Fig. 1B). This integrative multi-database strategy enhanced target coverage while reducing false-positive predictions, thereby providing a reliable basis for subsequent bioinformatic and functional analyses.

### Identification of Co-expression modules and differentially expressed genes

**Fig. 2:**
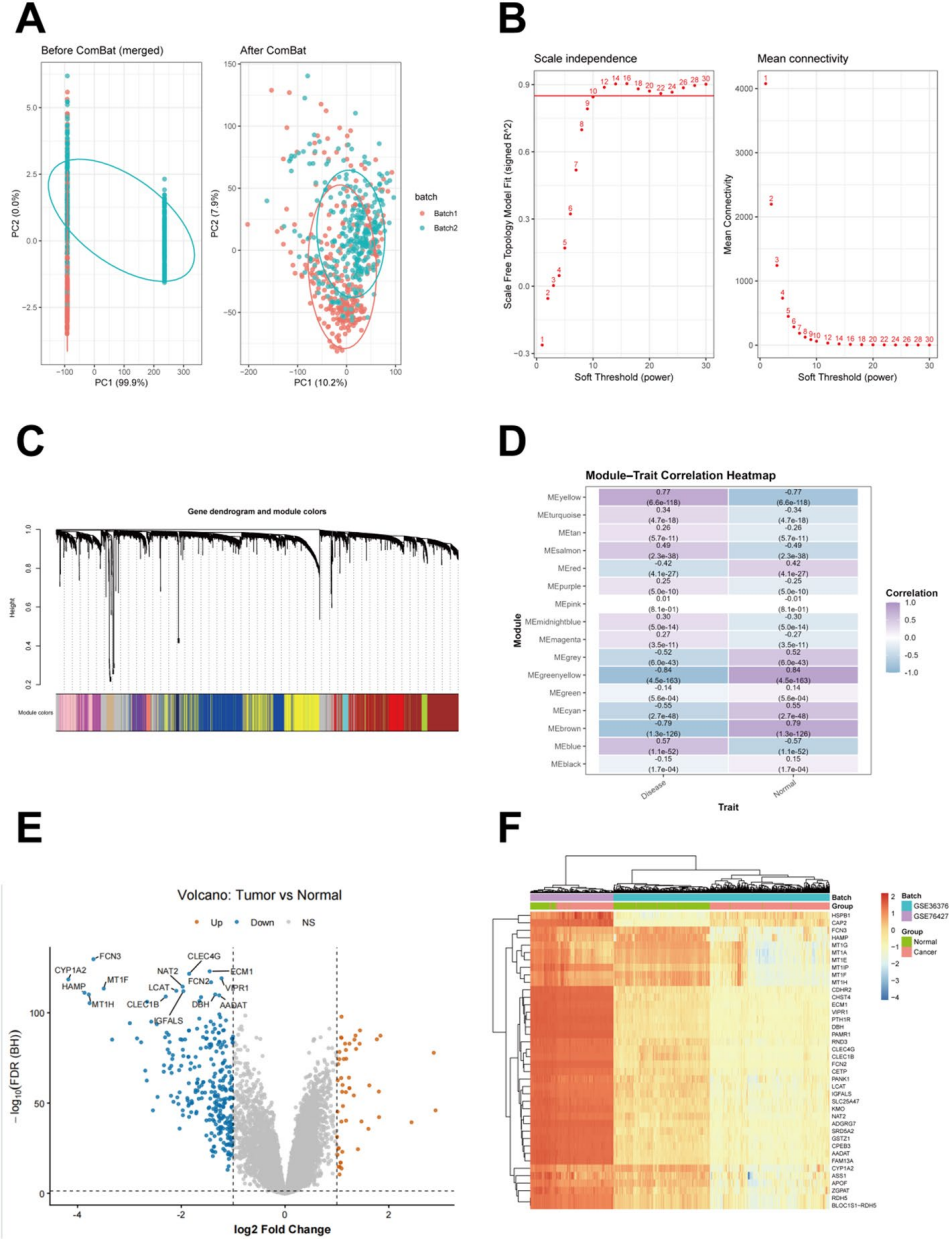
Identification of Disease-Associated Gene Modules and DEGs in HCC. **A** PCA before and after ComBat batch-effect correction, showing improved mixing of samples from different datasets after correction. **B** Selection of the soft-thresholding power in WGCNA was guided by the scale-free topology model fit (signed R²) and mean connectivity evaluated across powers 1–30. **C** Hierarchical clustering of the topological overlap matrix (TOM) showing module division. **D** Module–trait correlation heatmap (*P* < 0.05). **E** Volcano plot showing 288 DEGs with |logFC| > 1 and p.adj < 0.05 **F** Hierarchical clustering heatmap of the top 40 DEGs

To characterize transcriptomic differences between tumour and normal tissues, principal component analysis (PCA) was first performed. Prior to batch-effect correction, samples from different datasets were clearly separated in the principal component space. After correction using the ComBat method, samples from different batches were well mixed along the PC1 and PC2 axes, indicating improved data comparability and overall consistency (Fig. 2A). To identify gene modules significantly correlated with HCC phenotypes, Weighted Gene Co-expression Network Analysis (WGCNA) was conducted. A soft-thresholding power of β = 10 was determined as optimal to achieve a scale-free topology (R² ≥ 0.8; Fig. 2B). Hierarchical clustering using this parameter identified multiple co-expression modules, each represented by a distinct branch in the clustering dendrogram (Fig. 2C). Module–trait relationship analysis revealed that several modules were associated with disease status. Among them, the MEgreenyellow module, comprising 128 genes, exhibited the strongest negative correlation with Disease and a corresponding positive correlation with Normal status (Fig. 2D). In parallel, differential expression analysis was performed between tumour and normal tissues using |log₂FC| ≥ 1 and *p* < 0.05 as the cutoff criteria. A total of 288 differentially expressed genes (DEGs) were identified, including both significantly upregulated and downregulated genes, which were clearly visualized in the volcano plot (Fig. 2E). Hierarchical clustering based on these DEGs further demonstrated a clear separation between tumour and normal samples, confirming distinct global expression patterns consistent with sample groupings (Fig. 2F).

### Enrichment analysis

**Fig. 3:**
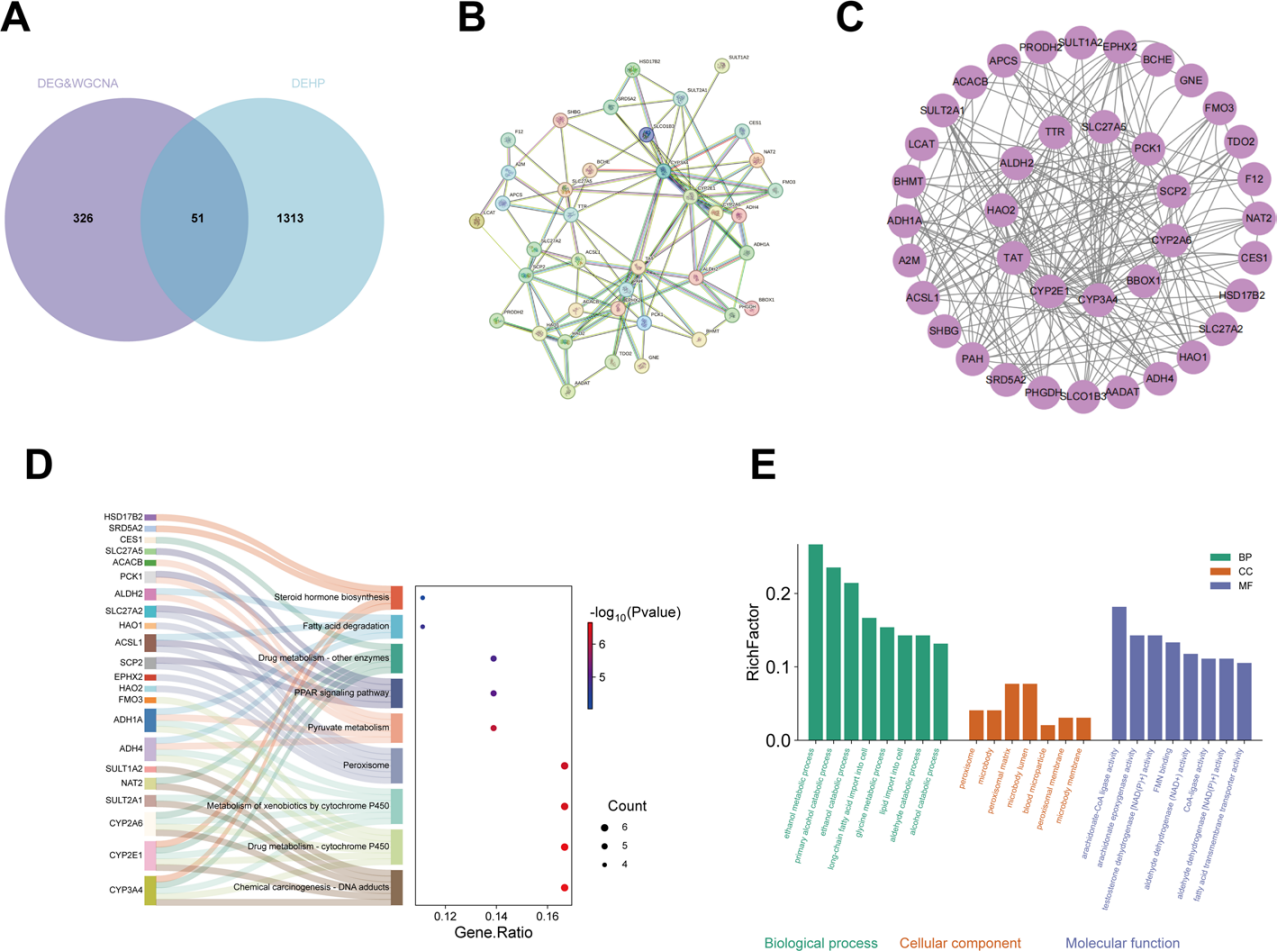
Overlap and Enrichment Analysis of DEHP and HCC Targets. **A **Venn diagram showing 51 shared targets. **B**, **C** Protein–protein interaction (PPI) network of the overlapping genes and key hub genes. **D** Chord diagram of the top enriched KEGG pathways for the 39 core genes. **E** Bubble chart of the top enriched GO terms

To further explore the potential molecular mechanisms underlying the role of DEHP exposure in disease initiation and progression, differentially expressed genes (DEGs) were integrated with key module genes identified by weighted gene co-expression network analysis (WGCNA) that were significantly associated with the disease phenotype (Fig. 3A). By intersecting the DEGs with the WGCNA-derived key module genes, a total of 51 overlapping genes were identified, which were considered potential candidate genes mediating the association between DEHP exposure and disease-related phenotypes. To further investigate the functional interactions among these candidate genes at the protein level, a protein–protein interaction (PPI) network was constructed using the STRING database. An interaction confidence threshold was applied to ensure the reliability of the network. During this process, genes without any known or predicted interactions (i.e., isolated nodes) were excluded from the network. As a result, 39 genes were retained for subsequent analysis, forming a PPI network comprising 39 nodes and 208 interaction edges (Fig. 3B–C). The high density of interactions and complex network architecture indicate that these proteins may function in a coordinated manner and collectively participate in DEHP-associated biological processes. Subsequently, Kyoto Encyclopedia of Genes and Genomes (KEGG) pathway enrichment analysis was performed based on the 39 key genes derived from the PPI network (Fig. 3D). The results revealed that these genes were significantly enriched in multiple pathways closely related to metabolism and xenobiotic processing, including chemical carcinogenesis–DNA adducts, drug metabolism–cytochrome P450, metabolism of xenobiotics by cytochrome P450, drug metabolism–other enzymes, fatty acid degradation, steroid hormone biosynthesis, PPAR signaling pathway, pyruvate metabolism, and peroxisome. Collectively, these enriched pathways highlight the involvement of xenobiotic metabolism, lipid metabolic reprogramming, and energy metabolism regulation, suggesting that DEHP may contribute to disease development and progression through disruption of these critical metabolic pathways. Furthermore, Gene Ontology (GO) functional enrichment analysis was conducted for the same set of 39 genes (Fig. 3E). In the Biological Process (BP) category, the genes were predominantly enriched in metabolism-related processes, including ethanol metabolic process, primary alcohol catabolic process, ethanol catabolic process, aldehyde catabolic process, alcohol catabolic process, long-chain fatty acid import into cell, and lipid import into cell. In the Cellular Component (CC) category, significant enrichment was observed in peroxisome, microbody, peroxisomal matrix, microbody membrane, and peroxisomal membrane. In the Molecular Function (MF) category, the enriched terms were mainly associated with aldehyde dehydrogenase (NAD⁺/NAD(P)⁺) activity, fatty acid transmembrane transporter activity, CoA-ligase activity, arachidonate-CoA ligase activity, arachidonate epoxygenase activity, and FMN binding.

### Integrated feature selection using multiple machine learning algorithms

**Fig. 4:**
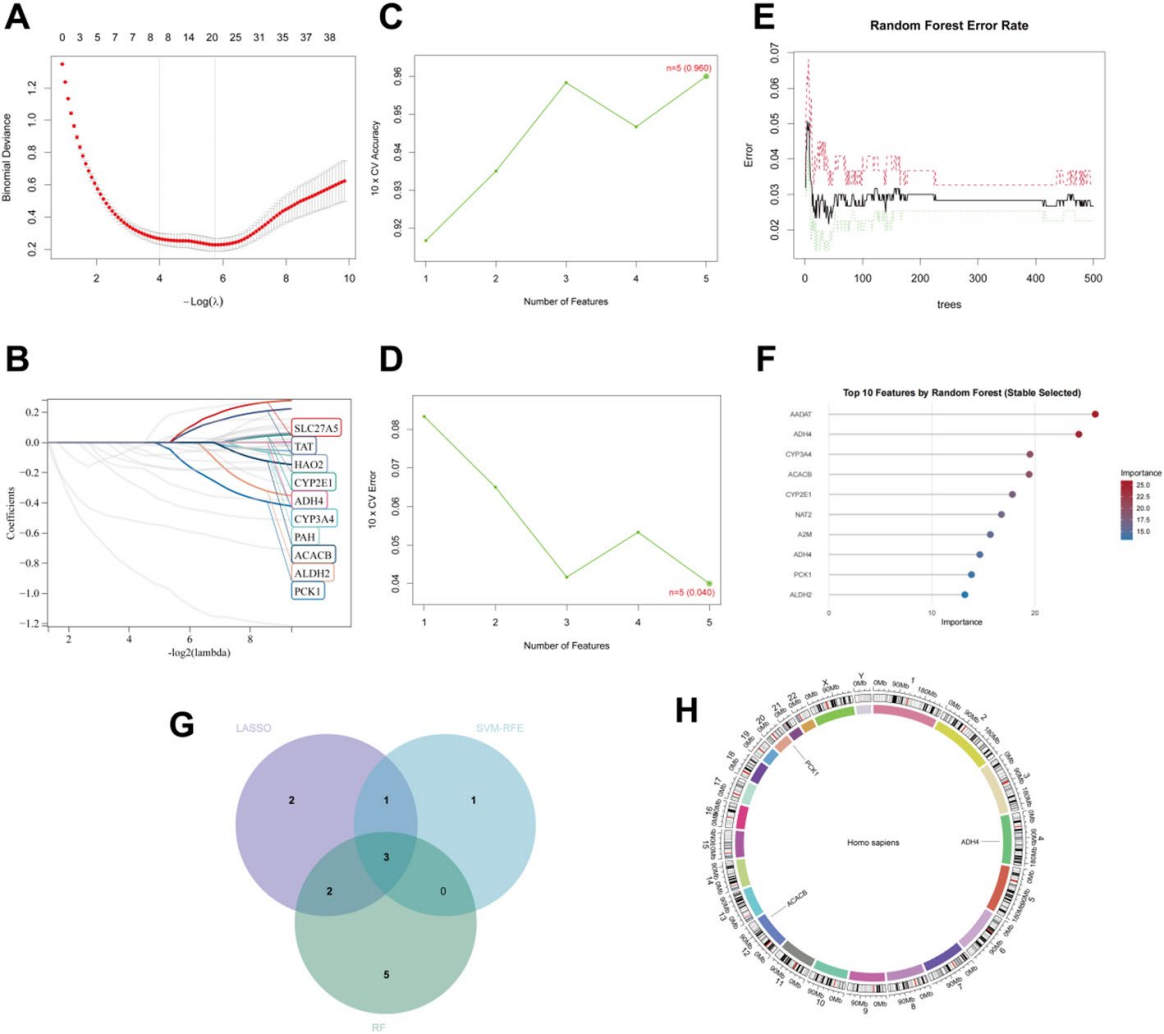
Machine learning and the validation of HCC-Related Genes. **A** LASSO cross-validation curve. **B** LASSO coefficient paths. **C** Plot depicting the relationship between feature number and 10-fold cross-validation accuracy; **D** Plot depicting the relationship between feature number and 10-fold cross-validation error. **E** Plot illustrating the relationship between random forest error and number of trees. **F** Feature importance plot. **G** Venn diagram combining results from three methods **H** Circle graphs showed the chromosomal locations of the HCC-Related Genes

To rigorously identify the most informative hub genes from the 39 overlapping candidates, three complementary machine learning algorithms were applied. LASSO regression with 10-fold cross-validation identified the optimal penalty parameter λ (Fig. 4A–B), which selected a subset of features with non-zero coefficients while eliminating redundant predictors. SVM-RFE iteratively removed features with the lowest ranking weights, and the relationship between feature number and cross-validation accuracy (Fig. 4C) as well as cross-validation error (Fig. 4D) demonstrated that optimal classification performance was achieved with a minimal feature set. Random Forest analysis using 500 decision trees showed rapid convergence of the out-of-bag error rate, indicating stable model performance (Fig. 4E). Variable importance ranking from the Random Forest model (Fig. 4F) further prioritized genes with the highest contribution to sample classification. Integration of the feature selection results from all three algorithms revealed a consensus set of three genes—ACACB, ADH4, and PCK1—that were consistently selected by LASSO, SVM-RFE, and Random Forest (Fig. 4G). Chromosomal localization analysis (Fig. 4H) showed that these three hub genes are distributed across different chromosomes.

**Fig. 5:**
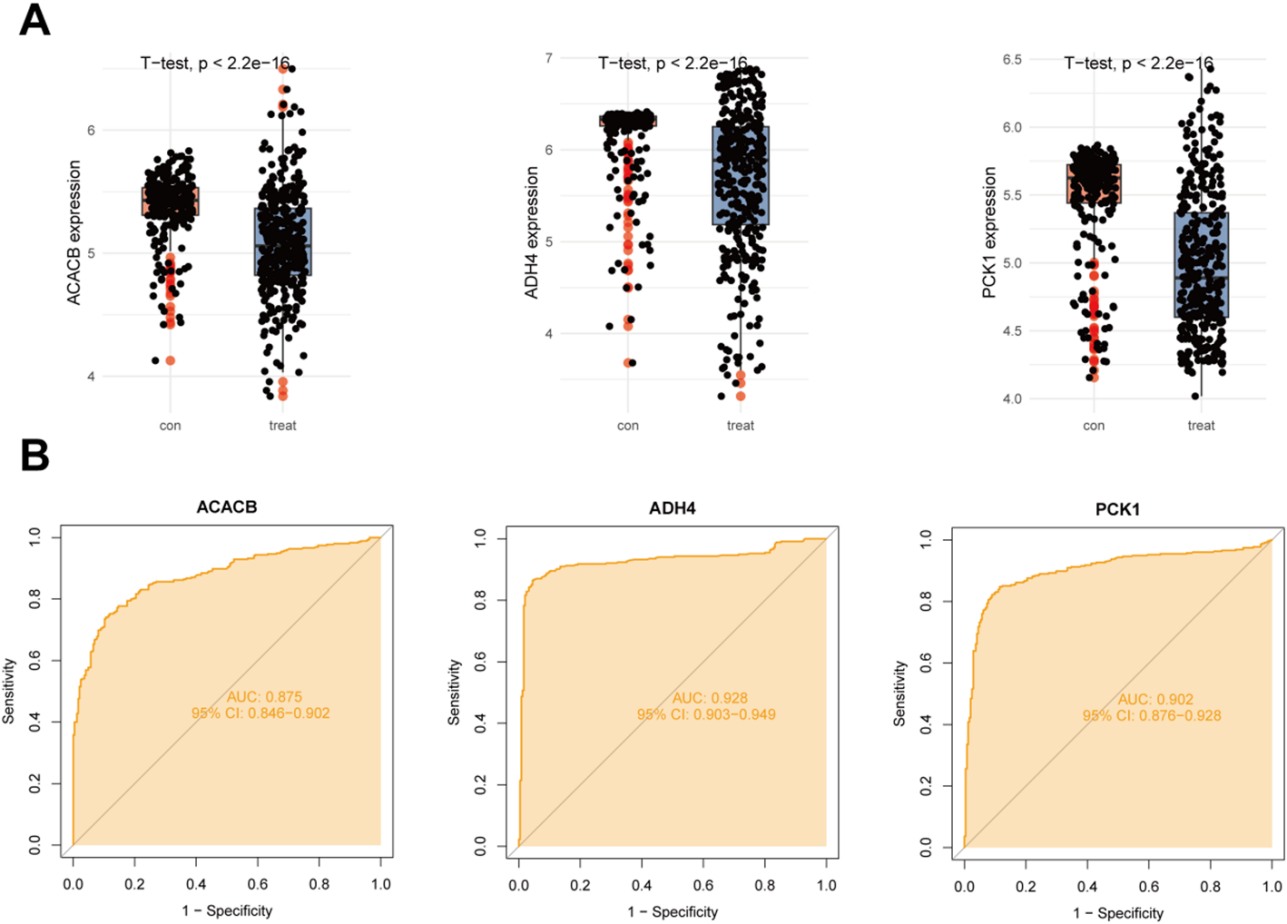
Differential expression (**A**) and ROC curve analysis (**B**) of ACACB, ADH4, and PCK1 between treatment and control groups. All three genes showed significant expression differences and strong discriminatory performance as indicated by AUC values

Differential expression analysis revealed that ACACB, ADH4, and PCK1 were significantly dysregulated between the treatment and control groups (Fig. 5A). Specifically, all three genes showed marked expression differences between groups (Student’s t-test, *p* < 2.2 × 10⁻¹⁶), with clear separation of expression distributions illustrated by boxplots and overlaid individual data points, indicating consistent and robust group-specific expression patterns. To further evaluate their discriminatory ability, ROC curve analysis was performed (Fig. 5B). ACACB demonstrated good predictive performance with an AUC of 0.875 (95% CI: 0.846–0.902), while ADH4 exhibited excellent diagnostic accuracy with the highest AUC of 0.928 (95% CI: 0.903–0.949). PCK1 also showed strong classification capability, achieving an AUC of 0.902 (95% CI: 0.876–0.928). Collectively, these results suggest that ACACB, ADH4, and PCK1 not only display significant differential expression but also possess substantial diagnostic potential for distinguishing treated samples from controls.

### Immune infiltration analysis

**Fig. 6:**
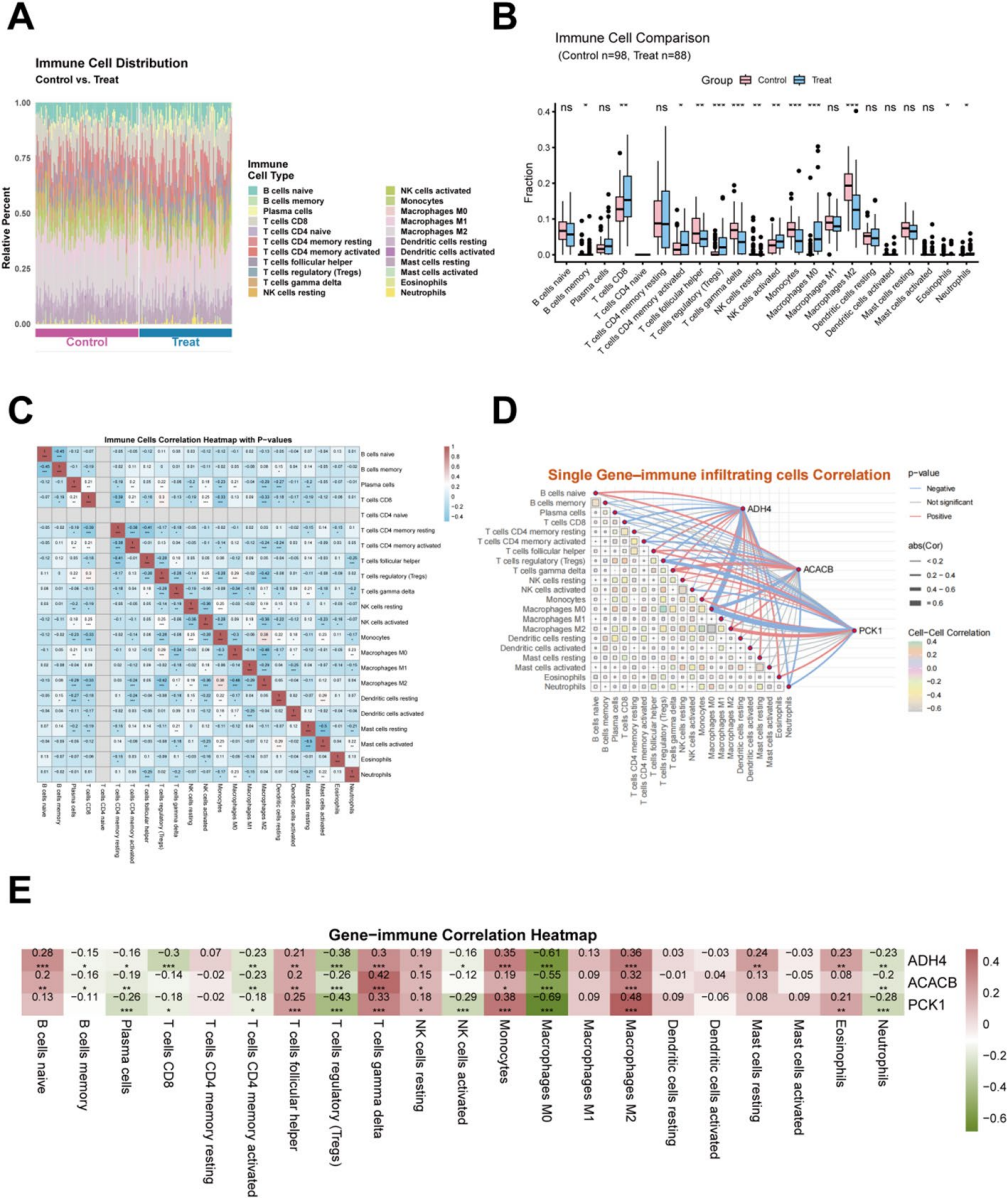
Immune infiltration analysis results **A** Stacked bar plot of immune cell infiltration. **B** Boxplot of immune cell infiltration. **C** Heatmap showing correlation coefficients between immune cell types, with red indicating positive correlations, blue indicating negative correlation, and color intensity reflecting the magnitude of the coefficient. **D** Correlation network based on mantel test. *p < 0.05, **p < 0.01, ***p < 0.001, **** *p* < 0.0001. **E** Correlation heatmap between gene expression and immune cell infiltration

To characterize the immune landscape associated with the studied condition, the CIBERSORT algorithm was used to estimate the relative proportions of 22 immune cell subsets in Control and Treat samples (Fig. 6A). Comparative analysis revealed significant differences in immune cell composition between the Control and Treat groups (Fig. 6B). Several lymphoid and myeloid cell populations exhibited significant alterations, whereas others remained comparable, indicating selective remodeling of the immune microenvironment. Pairwise correlation analysis among the 22 immune cell types revealed a complex network of positive and negative associations (Fig. 6C), with multiple immune cell pairs showing statistically significant correlations, suggesting coordinated immune infiltration patterns. The relationships between immune infiltration and the key genes ADH4, ACACB, and PCK1 were further explored. These genes showed significant correlations with multiple immune cell subsets, displaying distinct and heterogeneous immune association profiles (Fig. 6D). Consistently, the gene–immune correlation heatmap demonstrated that all three genes were strongly negatively correlated with macrophage M2 infiltration, while exhibiting variable associations with macrophage M0/M1 and specific lymphocyte subsets (Fig. 6E). Correlations with dendritic cells and mast cells were relatively weak, further highlighting the close link between key gene expression and macrophage-related immune features.

### Molecular docking analysis

**Fig. 7:**
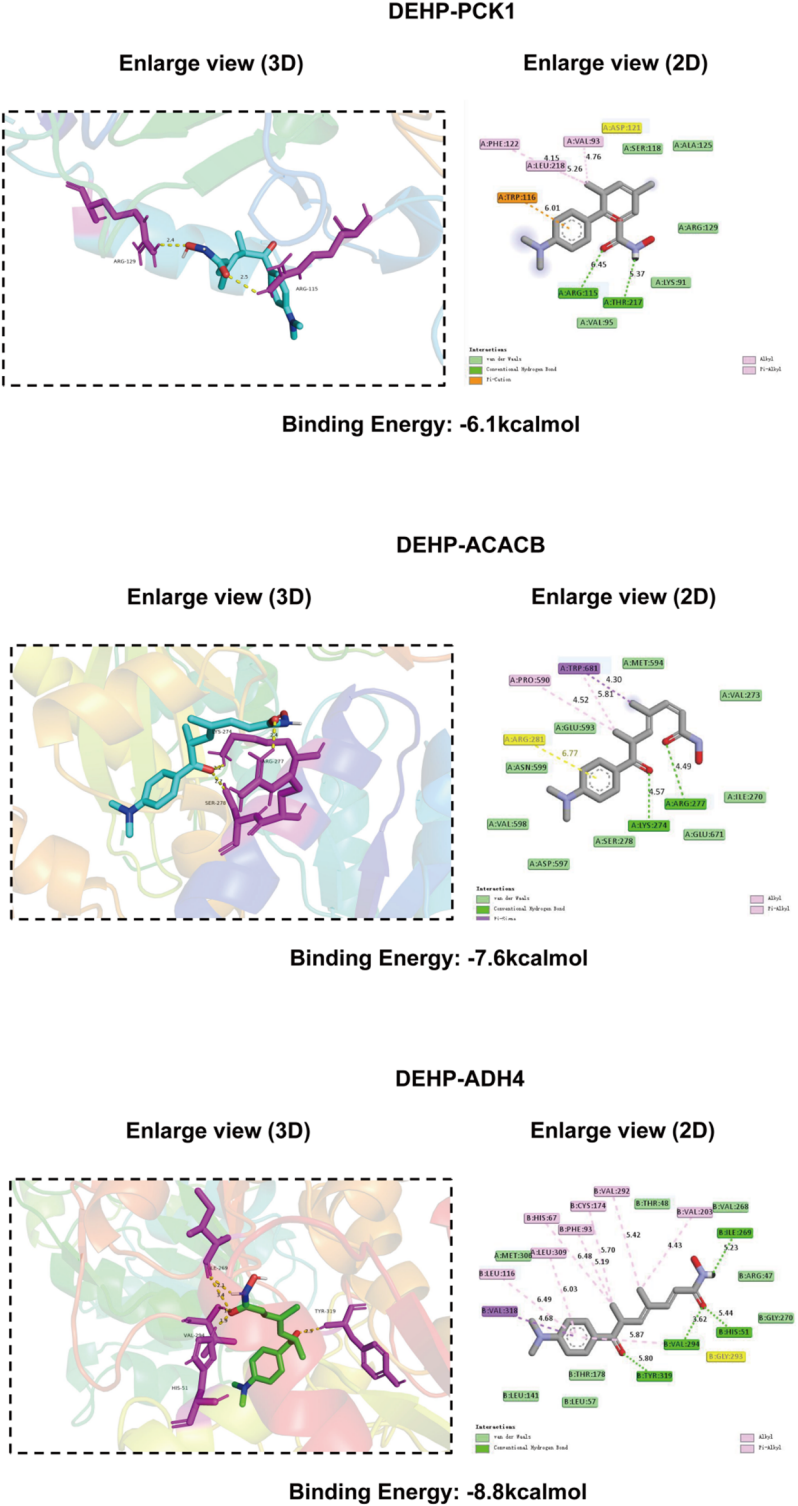
Molecular docking poses and binding free energies between DEHP and the three core target proteins

Molecular docking analysis indicated that PET could stably bind to ACACB, ADH4, and PCK1. The binding free energy of PET with ACACB was − 7.6 kcal/mol, mainly mediated by hydrogen bonds and hydrophobic interactions with key residues including ARG277, LYS274, and SER278. Among the three proteins, PET exhibited the strongest binding affinity toward ADH4, with a binding free energy of − 8.8 kcal/mol, forming extensive interactions with residues such as VAL294, ARG47, HIS51, and TYR319. In contrast, PET showed a relatively weaker interaction with PCK1, with a binding free energy of − 6.1 kcal/mol, primarily involving residues ARG115, LYS91, and THR217 through hydrogen bonding and hydrophobic interactions. Overall, PET displayed the highest binding affinity for ADH4, followed by ACACB and PCK1 (Fig.7).

## Discussion

Our integrative bioinformatics analysis suggests three metabolic enzymes—ACACB, ADH4, and PCK1—as candidate molecular links between DEHP exposure and hepatocellular carcinoma (HCC) development warranting experimental investigation. These computational predictions are consistent with accumulating experimental evidence that DEHP-induced hepatocarcinogenesis involves oxidative stress, epigenetic alterations, mitochondrial dysfunction, and disruption of lipid and energy metabolism. Pathway enrichment analyses highlighting xenobiotic metabolism, fatty acid degradation, and glucose metabolism dysregulation align with experimental studies showing that DEHP induces oxidative stress–mediated mitochondrial injury and metabolic disorders in the liver. The observed coordinated downregulation of ACACB, ADH4, and PCK1 in HCC tissues suggests a critical metabolic reprogramming pattern that may represent a mechanism through which DEHP exposure could facilitate tumor initiation and progression, consistent with the established role of metabolic shifts in cancer development.

ACACB encodes acetyl-CoA carboxylase beta (ACC2), a key regulator of fatty acid synthesis and oxidation through the production of malonyl-CoA [22]. Its downregulation in HCC underscores the complex and context-dependent roles of ACC enzymes in hepatocarcinogenesis [23, 24]. While genetic inhibition of ACC activity has been associated with increased susceptibility to liver tumorigenesis in some models, other studies suggest that ACC-mediated lipogenesis is required to maintain intracellular lipid homeostasis and prevent cell death [25]. This apparent paradox likely reflects isoform-specific functions and stage-dependent metabolic demands during HCC progression [26]. Notably, DEHP is known to activate peroxisome proliferator-activated receptor alpha (PPARα), a central regulator of lipid metabolism, suggesting that DEHP-induced dysregulation of ACACB may promote hepatocarcinogenesis through altered lipid homeostasis, oxidative stress, and mitochondrial dysfunction [27, 28].

Among the three hub genes, ADH4 exhibited the strongest diagnostic performance in distinguishing HCC from normal tissues. ADH4 plays a pivotal role in retinol metabolism by catalyzing the oxidation of retinol to retinoic acid, and its marked downregulation in HCC was significantly associated with poor prognosis. Mechanistically, ADH4 suppression may contribute to hepatocyte apoptosis, liver fibrosis, and tumor initiation, while impaired retinoic acid synthesis can reduce RAR/RXR-mediated expression of Wnt/β-catenin pathway inhibitors such as WIF-1, thereby facilitating oncogenic signaling [29]. In addition, given ADH4’s involvement in ethanol metabolism and xenobiotic detoxification, its downregulation may compromise hepatic capacity to metabolize DEHP and its toxic metabolite MEHP, which exhibits genotoxic and carcinogenic properties. Reduced detoxification capacity may exacerbate DEHP-induced apoptosis mediated by calcium overload, ROS-driven mitochondrial depolarization, and ERK/NF-κB signaling [30, 31].

PCK1, the rate-limiting enzyme of hepatic gluconeogenesis, was also significantly downregulated in HCC, consistent with the suppression of gluconeogenesis and preference for aerobic glycolysis characteristic of the Warburg effect [32]. Extensive evidence supports a tumor-suppressive role for PCK1 in HCC, as its overexpression inhibits proliferation, migration, and promotes apoptosis [33]. Mechanistically, PCK1 deficiency enhances tumor growth through AMPK inactivation, suppression of p27^Kip1, and activation of the CDK/Rb/E2F pathway, while restoration of PCK1 induces oxidative stress and apoptosis via metabolic reprogramming [34]. DEHP may contribute to PCK1 suppression through PPARα-mediated transcriptional regulation and by creating a metabolic environment favoring glycolysis through mitochondrial dysfunction and glucose metabolism disorder [35].

Immune infiltration analyses revealed that ACACB, ADH4, and PCK1 display distinct correlations with multiple immune cell populations, suggesting that DEHP-induced metabolic reprogramming may reshape the tumor immune microenvironment. Alterations in lipid and glucose metabolism can profoundly affect immune cell fitness and function, thereby facilitating immune evasion. Dysregulation of ACC activity influences fatty acid metabolism in T cells, affecting their energy maintenance and anti-tumor capacity under tumor microenvironment stress [36]. Similarly, impaired retinoic acid production resulting from ADH4 downregulation may disrupt immune regulation [37, 38], while enhanced glycolysis and nutrient competition associated with PCK1 deficiency may suppress immune cell function while supporting malignant cell survival [39–41].

Molecular docking analyses further demonstrated that DEHP can stably bind all three hub proteins, with the strongest affinity observed for ADH4 (binding energy: −8.8 kcal/mol), providing structural support for potential direct protein–ligand interactions. Although ACACB and PCK1 exhibited moderate binding energies (− 7.6 and − 6.1 kcal/mol, respectively), such interactions may still contribute to hepatotoxic effects under chronic exposure conditions, given the prolonged and cumulative nature of environmental DEHP exposure in human populations. Collectively, these findings suggest that ACACB, ADH4, and PCK1 function as both putative DEHP-responsive targets and critical regulators of HCC-associated metabolic and immune alterations, highlighting their potential dual roles as biomarkers and therapeutic targets. The strong diagnostic performance of these genes, particularly ADH4 with an AUC of 0.928, underscores their potential utility for early HCC detection or risk stratification, especially in populations with documented high DEHP exposure levels. From a public health perspective, this study provides mechanistic support for epidemiological observations linking widespread environmental DEHP exposure to adverse liver outcomes, including non-alcoholic fatty liver disease and hepatocellular carcinoma, thereby reinforcing the need for continued regulatory efforts to minimize human exposure to phthalates.

Nevertheless, several important limitations must be acknowledged. First, this study employed entirely computational approaches without experimental validation, precluding direct causal inference between DEHP exposure and the observed molecular alterations. Second, the transcriptomic datasets analyzed derived from HCC patients with diverse etiologies, and DEHP exposure status was unknown, limiting direct exposure-outcome associations. Third, the molecular docking simulations used rigid protocols that do not fully capture protein flexibility or account for MEHP, the primary bioactive DEHP metabolite. Fourth, immune cell proportions estimated from bulk RNA-sequencing may not reflect the spatial organization or functional states revealed by single-cell approaches. Future research should prioritize: (1) experimental validation of predicted DEHP–protein interactions through enzyme kinetic assays and structural studies; (2) dose-response assessment at environmentally relevant exposure levels; (3) prospective epidemiological studies with quantified phthalate biomarkers to establish causality; and (4) clinical evaluation of ACACB, ADH4, and PCK1 as early detection biomarkers in populations with documented DEHP exposure.

## Conclusions

This integrative computational study suggests ACACB, ADH4, and PCK1 as candidate molecular links between DEHP exposure and hepatocellular carcinoma through metabolic reprogramming and immune microenvironment remodeling. These three enzymes, which regulate lipid metabolism, xenobiotic detoxification/retinol metabolism, and gluconeogenesis respectively, show significant downregulation in HCC and strong diagnostic performance (AUC: 0.875–0.928), with ADH4 demonstrating the highest discriminatory capacity. Computational docking analyses indicating favorable DEHP binding to all three proteins, combined with their significant correlations with immune cell infiltration patterns, provide testable hypotheses whereby DEHP-induced dysregulation of these metabolic nodes could promote hepatocarcinogenesis by disrupting lipid homeostasis, impairing detoxification capacity, enhancing glycolytic metabolism, and facilitating immune evasion. If experimentally validated, these findings could establish ACACB, ADH4, and PCK1 as biomarkers for early detection of DEHP-associated hepatic dysfunction, inform mechanistic understanding of phthalate hepatotoxicity, and identify potential therapeutic intervention points. From a public health perspective, this study provides computational support for epidemiological concerns linking widespread DEHP exposure to adverse liver outcomes, reinforcing the need for continued regulatory efforts while future research establishes causal relationships through rigorous experimental validation, including enzyme kinetic assays, controlled exposure studies, and prospective clinical investigations.

## Supplementary Information

Below is the link to the electronic supplementary material.

Supplementary Material 1.

## Data Availability

The datasets analyzed in this study are available in the NCBI Gene Expression Omnibus (GEO) repository under accession numbers GSE36376 and GSE76427. The DEHP target prediction data were obtained from PubChem (https://pubchem.ncbi.nlm.nih.gov/), ChEMBL (https://www.ebi.ac.uk/chembl/), PharmMapper (http://www.lilab-ecust.cn/pharmmapper/), and SwissTargetPrediction (http://www.swisstargetprediction.ch/). The protein-protein interaction data were obtained from the STRING database (https://string-db.org/). All other data generated or analyzed during this study are included in this published article and its supplementary information files.
