# Supplementary material for: Mechanism exploration of di(2-ethylhexyl) phthalate (DEHP)-induced hepatocellular carcinoma via network toxicology and molecular docking analysis
Source: Discov Oncol. 2026 Apr 24;17:807. doi: 10.1007/s12672-026-05081-2 (PMC13216419; doi:10.1007/s12672-026-05081-2)
Supplement: Supplementary file 1 — Supplementary Material 1. [file 12672_2026_5081_MOESM1_ESM.docx]

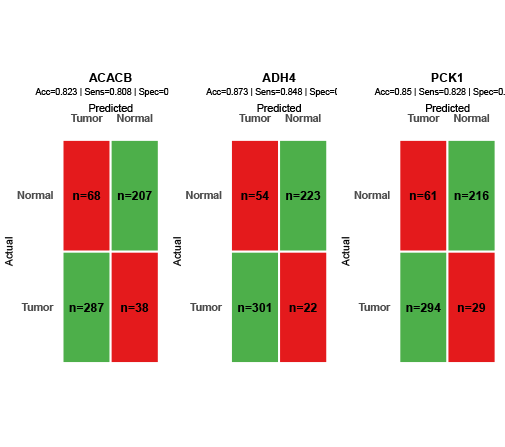


**Supplementary** **Figure S1** . Confusion matrices for ACACB, ADH4, and PCK1. Confusion matrices showing classification performance of each hub gene in distinguishing hepatocellular carcinoma (HCC) tumor tissues (n = 355) from adjacent normal liver tissues (n = 245). For each gene, the optimal classification threshold was determined by Youden's index based on ROC analysis. True positives (TP), false positives (FP), true negatives (TN), and false negatives (FN) are shown within each matrix. Performance metrics (accuracy, sensitivity, specificity) are provided below each plot. (A) ACACB: accuracy = 0.823, sensitivity = 0.808, specificity = 0.845. (B) ADH4: accuracy = 0.873, sensitivity = 0.848, specificity = 0.910. (C) PCK1: accuracy = 0.850, sensitivity = 0.828, specificity = 0.882.
